# Evaluating the effect of a herb on the control of blood glucose and insulin-resistance in patients with advanced type 2 diabetes (a double-blind clinical trial)

**DOI:** 10.22088/cjim.11.1.12

**Published:** 2020

**Authors:** Mahmoud Parham, Mohammad Bagherzadeh, Majid Asghari, Hossein Akbari, Zahra Hosseini, Maryam Rafiee, Jamshid Vafaeimanesh

**Affiliations:** 1 Gastroenterology & Hepatology Disease Research Center, Qom University of Medical Science, Qom, Iran; 2 Clinical Research Development Center, Shahid Beheshti Hospital, Qom University of Medical Sciences, Qom, Iran; 3 Department of Traditional Medicine, Qom University of Medical Sciences, Qom, Iran; 4 Department of Epidemiology, Kashan University of Medical Sciences, Kashan, Iran; 5 Department of Radiology Kashan University of Medical Sciences, Kashan, Iran; 6 Department of Obstetrics and Gynecology, Kashan University of Medical Sciences, Kashan, Iran

**Keywords:** Type 2 diabetes, Herbal medicine, Insulin-resistance

## Abstract

**Background:**

Different benefits of various herbal medicines in decreasing blood sugar have been reported in different clinical trials so far. Considering the growing tendency toward these combinations and the booming market, inappropriate advice is growing accordingly. Hence, it is necessary to evaluate the effects and possible complications of such combinations on health status and blood glucose control.

**Methods:**

Two 38-subject groups were formed and a 12-week treatment program was administered for both groups. The inclusion criteria were failure to control blood glucose with two oral medicines, unwillingness to inject insulin. The medicine was prepared in capsules by Booali Company. Each capsule weighed 750 mg and contained nettle leaf 20% (w/w), berry leaf 10% (w/w), onion and garlic 20% (w/w), fenugreek seed 20% (w/w), walnut leaf 20% (w/w), and cinnamon bark 10% (w/w) all in powder.

**Results:**

At the beginning of the study, there was no significant difference between the subjects regarding the evaluated parameters, but after the intervention, the level of glucose was significantly lower in fasting (P=0.0001) and 2-hour postprandial (P=0.002) levels. The level of glycated hemoglobin A1c (HbA1c) (P=0.0001) also decreased from 0.33±9.72 % to 0.20±8.39 %. Finally, the level of insulin resistance reduced from 1.9±4.1 to 1.4±2.6 (P=0.001) after consuming herbal medicine.

**Conclusion:**

According to the results of the current study, the herbal combination was effective in controlling blood sugar, and considering the reduction of HbA1c by 1.31 %, it seems that the herbal combination is an effective medicine to treat diabetes.

Type 2 diabetes is one of the global public health concerns in the 21st century; both the developed and the developing countries are experiencing increasing rates of diabetes (1). Indeed, the prevalence and incidence of diabetes is still increasing constantly indicating a global diabetes epidemic (2). Currently, there are more than 150 million people with diabetes worldwide and it is predicted that the number increases to 366 million (constituting 4.4% of the world population) by 2030 (3). Diabetes is now a main cause of disability and hospitalization of patients and imposes a significant financial burden to the community. In India, 92 million USD is spent to treat patients with diabetes every year (4). In the first step, diabetes is treated by an oral antidiabetic drug (OAD); but with further development of the disease, the patient needs one or more antidiabetic drugs, in addition to the first prescribed agent (5). Before the discovery of insulin and invention of chemical drugs, physicians used herbal medicines to control diabetes.

Considering the high prevalence of diabetes and the concerns over the consequences of its complications, a wide range of complementary and alternative medicines (CAMs) with different effectiveness is used to control diabetes. Patients with diabetes show interest in using complementary and alternative medicine to control their blood sugar 1.6 times more than their non-diabetic counterparts (6). Accordingly, 2-3.6 million Americans rely on complementary and alternative medicines to treat their diabetes (7). Reasons such as complicated common therapeutic regimens, hypoglycemia, personal beliefs, and side effects of drugs have decreased the patients’ compliance with common treatments and encouraged them to use complementary treatments (7). The tendency toward CAMs to treat diabetes has increased considerably from 30% to 57% (8). In 2013, the estimation of spending on complementary and integrative medicine (CIM) in the USA reached 34 billion USD (9). 

The efficiency and benefits of more than 1200 herbal medicines in reducing blood sugar and diabetes complications have been reported so far (6). Considering the daily increase of interest in using such combinations and the booming market, inappropriate advice is growing in parallel. Hence, more meticulous studies should be conducted on the effectiveness and possible complications of such combinations on health status and control of blood sugar. Therefore, the current study aimed to evaluate the effect of a herbal medicine capsule on the control of blood glucose and insulin resistance. This capsule contained Urtica Dioica leaf, Morus Alba leaf, Allium sativum powder, Trigonella foenum-graecum seeds, Juglans regia leaf, and Cinnamomum zeylanicum bark. This drug combination is a capsule with a manufacturing license from the Iranian Ministry of Health Food and Drug Administration (License number: 026-89-S-T). Many studies have been conducted on some of the components of this drug, but so far no clinical trial study has been performed on the entire drug combination. A number of studies are as follows:

Rajarajeswari et al. indicated that the aqueous extract of Fenugreek has significantly greater anti-diabetic effects as compared with the alcoholic extract (10, 11). Compound GІІ in Fenugreek can reduce the blood glucose and hemoglobin glycosylated levels while elevating the insulin level (12). According to Divbandand. K et al., oral administration of aqueous extract of Juglans Regia leaf at 400 mg/kg for 4 weeks significantly reduced the blood glucose level in diabetic rats (P=0.009) (13). Shihabudeen M. S et al. revealed that the administration of alcoholic extract of Cinnamomum Zeylanicum bark in rats fed with sucrose and maltose led to inhibition of α-glucosidase and could stop post-prandial hyperglycemia. They believed that Cinnamomum Zeylanicum extract, in a reversible process, controls the hyperglycemia induced by sucrose and maltose after meal through inhibiting α-glucosidase enzyme (14). As Gholipour et al. presented in their study about the effect of Urtica Dioica leaf on diabetes management, the administration of Urtica Dioica leaf before induction of diabetes in rats increased the production of β-cells in Langerhans islets and decreased the concentration of blood glucose in diabetic rats by 60% (15).

Regarding the growth in the prevalence of this disease and the increased use of herbal drugs and lack of knowledge about the effect of this herbal compound on diabetes, this study examined the level of blood glucose in diabetic patients in association with this herbal medicine.

## Methods

**Figure 1:**
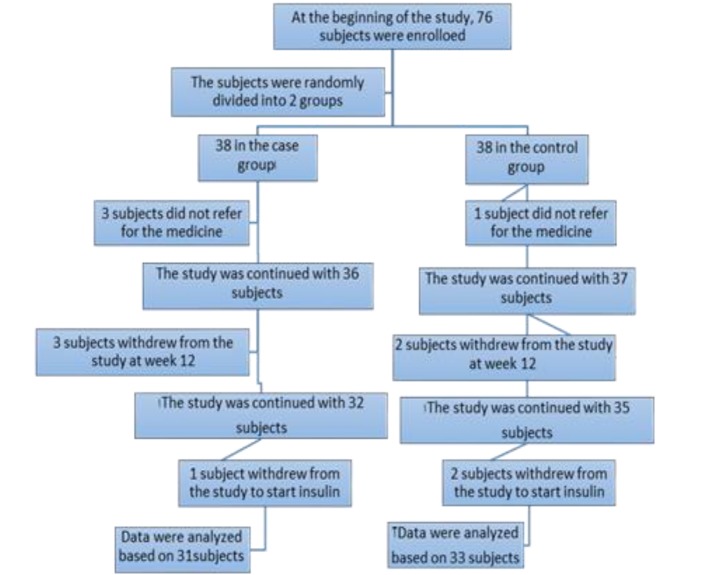
The Study Flowchart

**Study Project: **The current double-blind clinical trial was conducted on 76 patients with diabetes in two groups of case and control (placebo) in Shahid Beheshti Hospital, Qom, Iran, from 2014 to 2015. Figure 1 reveals the flowchart of the study.

**Study Population: **Since the mentioned herbal combination had not been used in any other human studies for controlling diabetes, the combination was not used in the initial and standard treatments of diabetes in the current study. Also, as the study did not aim to deprive patients with diabetes from standard treatments, patients with no other therapeutic choices constituted the study population. Hence, the population was selected from patients who had completed the treatment course of two OADs, but due to inefficiency of OADs, they were candidates for starting insulin and based on some personal reasons refused insulin injection, despite the emphasis of their physicians. 

**Inclusion and exclusion criteria: **The inclusion criteria were: having type 2 diabetes within the age range of 25-70 years, FBS > 130 mg/dL, and 2hppBG >180 mg/dL. The patients should have received two OADs and insulin injection, but were not willing to be injected. On the other hand, the exclusion criteria were: having cardiovascular, renal, and liver diseases, pregnancy, breastfeeding and history of allergy to herbal products, having diseases with increased risk of allergy such as asthma and atopy, allergy to milk, lactose intolerance, and participation in another clinical trial. 

**Sampling: **To determine the sample size in the current study, the sample size formula was used in two populations based on a previous study (16). Accordingly, the minimum sample size for each group was determined as 31, and based on estimating 15% attrition, eventually 38 subjects were assigned into each group.

**Random Sampling: **To select the subjects randomly, a box with 76 numbered cards (from 1 to 76) was prepared and the subjects included in the study took a card and referred to the project collaborator pharmacy. Based on the card number, a box of medicine was given to the subject; half of the boxes contained the medicine and the rest were placebo. 

**Requirements: **Both medicine and placebo boxes were prepared by the same company with similar packages and boxes for all subjects. Half of the boxes contained the medicine and the rest were placebo, which were codified by a person not involved in the study by giving a number to each box. After enrolment of the subject into the study, they were given a number, and based on that the project collaborator pharmacy gave them a box with the same number. The subject was followed-up until the end of the study based on that number. At the end of the study, the subjects were divided into the case and control groups based on their numbers.

**Interventions**

**Medicine: **The medicine was prepared in capsules by Booali Company. Each capsule weighed 750 mg and contained of nettle leaf 20% (w/w), berry leaf 10% (w/w), onion and garlic 20% (w/w), fenugreek seed 20% (w/w), walnut leaf 20% (w/w), and cinnamon bark 10% (w/w) all in powder. 

**Placebo: **The placebo was also prepared by the same company which manufactured the medicine in similar packages; each capsule contained micro-crystalline cellulose 50% (Avesil) and bran 50%. The shape, weight, taste, and color of the placebo capsule were similar to those of the medicine. 

**The study period and grouping: **The study was conducted in a 12-week period and the case group was under the treatment with an herbal medicine (with a determined combination certified by the Iranian Ministry of Health and Medical Education), three times a day, each time one capsule in addition to the previous drugs. Similarly, the placebo group also received placebo three times a day, each time one capsule in addition to the previous drugs.

**Outcomes: **The morning fasting blood samples in the current study were taken from antecubital veins. Then, the samples were centrifuged and the serum was collected. Glucose, urea, creatinine, triglycerides, cholesterol, Aspartate Transaminase (AST), alanine transaminase (ALT), and alkaline phosphatase (ALP) were isolated by reagent kits in a stable liquid using enzymatic method. C-reactive protein (CRP) was measured by the quantitative method of CRP-Latex immunoturbidimetric Assay (CRP-LIA). To measure insulin and HbA1c, Bio-Rad and NycoCard™ HbA1c kits were used, respectively. The level of low density lipoprotein (LDL) cholesterol was calculated by Friedewald formula. The level of insulin-resistance was also measured based on HOMA-IR formula as follows: FBS (mg/dL) x serum insulin / 405. Blood pressure was recorded by a trained nurse using a mercury manometer, after 5-min resting at sitting position. 

**Side effects and reports: **During the study, the subjects were checked by the study expert every two weeks to monitor treatment and possible side effects through telephone call. Also, to report any side effects out of the mentioned time interval, the telephone number of the expert was given to the subjects. Note that at the beginning and end of the study, kidney and liver function tests were also performed.

**Ethical considerations: **The produced medicine was certified by Iranian Food and Drug Administration, and Ministry of Health and Medical Education (No. 85-026-s-t). The study protocol was provided based on Helsinki declaration on ethical principles and approved in the Research Ethics Committee of Qom University of Medical Sciences, Qom, Iran (ir.muq.rec.1395.42). The study was also designed based on customary, ethical, and religious norms of the associated community and data confidentiality was carefully followed by the study authors. The medicine and its probable side effects were thoroughly explained by a physician to the subjects. Written informed consent was obtained from all subjects. The subjects were free to withdraw from the study at any time they desired. Also, all laboratory tests and medicines were free of charge for the subjects. The study was registered in the Iranian Registry of Clinical Trials (IRCT2016011826097N1).

**Study analysis: **To analyze the qualitative variables, frequency and the percentage of frequency, and to analyze the quantitative variables, the mean and standard error of the mean (SEM).were measured, based on the collected data. To analyze quantitative variables further, the independent t-test, was used, and to measure parameter changes in the group, the paired samples t-test was employed. To analyze the qualitative variables further, the Mc Nemar Chi-square test was used. The impact of multivariate effects of the medicine was analyzed by covariance analysis. A P<0.05 was considered significant. 

## Results

In the current study, two groups of 38 subjects (the case and control) were evaluated. The subjects were followed up every two weeks by telephone call and every six weeks through physical examination (the study duration was 12 weeks). At the beginning of the study, three subjects did not refer to pharmacy for their medicines and were absent in follow-ups; one subject from the control group and two subjects from the case group. After 12 weeks of the study, five subjects were excluded from the study due to unwillingness and lack of cooperation with the study. No side effects and complications were reported from the subjects who consumed the medicine up to week 12. At the end of the study, three subjects (one from the case and two from the control groups) were excluded due to their willingness to use insulin. Out of 64 subjects, 33 subjects remained in the control and 31 in the case groups. There was no significant difference between the groups regarding the number of excluded subjects. According to the laboratory tests, there were no significant differences in the level of urea, creatinine, and amino transaminase enzymes between the groups. 

In the current study, 64 subjects were evaluated, including 16 (25%) males and 48 (75%) females, out of whom 33 (8 males and 25 females) subjects were in the case group and 31 (8 males and 23 females) were in the control group. The difference between the groups regarding the number of subjects was insignificant (P=0.8). 

The demographic information and medical data of the subjects at the beginning of the study are reported in table 1.

**Table 1:** The Information of subjects in both groups, before the intervention

Groups	**Control ** **(Mean±SEM)**	**Case** **(Mean±SEM)**	**P-value**
Age	56.9±1.48	56.51±1.47	0.840
Weight	77.24±2.53	76.58±1.84	0.836
Disease duration (year)	11.57±0.89	12.12±0.96	0.67
Systolic blood pressure	130.90±2.49	127.90±2.67	0.414
Diastolic blood pressure	84.24±1.32	82.41±1.08	0.295
2hppBG	291.45±16.43	318.12±14.08	0.225
FBS	192.72±14.37	210.61±9.29	0.307
HbA1c	9.26±0.34	9.72±0.33	0.346
Urea	31.00±1.45	30.00±1.62	0.650
Creatinine	0.92±0.02	0.98±0.02	0.201
Triglyceride	194.09±13.88	176.90±15.05	0.404
Cholesterol	195.18±6.24	187.00±6.87	0.381
LDL cholesterol	104.33±4.21	109.87±11.49	0.645
HDL cholesterol	45.27±1.81	45.12±1.72	0.954
SGOT	17.15±1.59	18.58±1.12	0.471
SGPT	21.03±2.32	24.22±2.37	0.340
ALP	119.18±13.20	129.58±10.59	0.544
CRP	2.21±0.35	2.29±0.36	0.888
HOMA-IR	3.3±2.1	4.1±1.9	0.1

2hppBS: 2-Hour Post-Prandial Blood Glucose; FBS: Fasting Blood Sugar; HbA1C: Glycated Hemoglobin A1c; LDL: Low-Density Lipoprotein; HDL: High-Density Lipoprotein; SGOT: Aspartate Transaminase; SGPT: Alanine Transaminase; ALP: Alkaline Phosphatase; CRP: C-Reactive Protein

According to table 1, there was no significant difference between the groups regarding the evaluated parameters. Based on table 2, consuming the medicine significantly reduced the level of FBS from 210.61±9.29 to 178.25±5.50 mg/dL. Also, the use of medicine significantly lowered the level of 2hpp BS (P=0.002). In addition, according to table 2, the use of medicine significantly reduced the level of HbA1c (P=0.0001). The level of glucose improved in the subjects through reducing insulin resistance; the level of insulin resistance diminished from 1.9±4.1 to 1.4±2.6 by consuming the medicine (P=0.001). 

However, the medicine had no effect on blood pressure and lipid profile values. The subjects’ data were collected after consuming the placebo and accordingly, no significant difference was observed on the evaluated parameters (table 2).

**Table 2:** The information of subjects in the case and control groups, before and after intervention

	**Control group**			**Case group**		
	**Before** **(Mean±SEM)**	**After** **(Mean±SEM)**	**p-value**	**Before** **(Mean±SEM)**	**After** **(Mean±SEM)**	**P-value**
Systolic blood pressure	130.90±2.49	129.09±2.45	0.61	127.90±2.67	124.06±2.77	0.68
Diastolic blood pressure	84.24±1.32	77.58±1.17	0.82	82.41±1.08	77.58±1.17	0.89
2hppBG	291.45±16.43	266.85±11.59	0.097	318.12±14.08	270.90±9.21	0.002
FBS	192.72±14.37	178.96±8.45	0.328	210.61±9.29	178.25±5.50	0.0001
HbA1c	9.26±0.34	8.66±0.28	0.078	9.72±0.33	8.39±0.20	0.0001
Urea	31.00±1.45	29.92±1.82	0.56	30.00±1.62	26.74±1.07	0.6
Creatinine	0.92±0.02	0.96±0.02	0.53	0.98±0.02	0.90±0.03	0.58
Triglyceride	194.09±13.88	212.48±15.53	0.168	176.90±15.05	213.38±16.79	0.078
Cholesterol	195.18±6.24	194.63±7.35	0.941	187.00±6.87	193.22±5.13	0.419
LDL cholesterol	104.33±4.21	112.93±3.59	0.126	109.87±11.49	128.90±9.54	0.270
HDL cholesterol	45.27±1.81	45.03±1.46	0.909	45.12±1.72	44.64±1.73	0.797
SGOT	17.15±1.59	20.51±1.02	0.68	18.58±1.12	21.38±1.35	0.70
SGPT	21.03±2.32	22.12±1.42	0.65	24.22±2.37	23.70±2.52	0.76
ALP	119.18±13.20	161.30±12.28	0.81	129.58±10.59	156.29±7.93	0.78
CRP	2.21±0.35	2.79±0.38	0.270	2.29±0.36	2.81±0.30	0.231
HOMA-IR	3.3±2.1	3.2±2	0.6	4.1±1.9	2.6±1.4	0.001

2hppBS: 2-Hour post-prandial blood glucose; FBS: fasting blood sugar; HbA1C: glycated hemoglobin A1c; LDL: low-density lipoprotein; HDL: high-density lipoprotein; SGOT: aspartate transaminase; SGPT: alanine Transaminase; ALP: alkaline phosphatase; CRP: C-Reactive Protein

## Discussion

Type 2 diabetes is a multifactorial disease associated with different complications such as obesity, hypertriglyceridemia, impaired glucose tolerance (IGT), and increased insulin resistance, imposing a heavy financial burden to the healthcare economy. Considering the high prevalence of this disease and the increasing tendency of people toward consuming herbal medicines, it is necessary to evaluate the effects of such medicines on controlling blood glucose and the associated side effects. Recent evaluations have suggested that more than 80% of people in developing countries use herbal medicines to treat different diseases (7). The current study evaluated the effectiveness of an herbal combination with no significant side effects on the control of blood glucose in a group of patients not eligible to receive OADs. Since there was no similar combination for the herbal medicine, its effects cannot be compared with the results of other studies. Therefore, every component of the medicine was evaluated individually. The herbal medicine used in the current study contained nettle leaf 20% (w/w), berry leaf 10% (w/w), onion and garlic 20% (w/w), fenugreek seed 20% (w/w), walnut leaf 20% (w/w), and cinnamon bark 10% (w/w), all in powder. Vengerovskii AI et al. (17) reported useful effects of nettle (100 mg/kg) on the control of blood glucose in diabetic rats. They also indicated triglyceride-lowering effects for nettle at the same dose. Kianbakht S.et al. evaluated the effects of 500 mg nettle three capsules a day for three months and reported positive therapeutic effects (18). According to their results, nettle has simulating effects on the secretion of insulin, peroxisome proliferator-activated receptor (PPAR)-gamma agonist, and the inhibitory effect on alpha-glucosidase. According to the results of the current study including elevated insulin and diminished 2hppBG and insulin resistance, the theory of Kianbakht S. et al. seems to be correct. Patel SSet al. studied the effect of essential oil of nettle leaf on diabetic rats and reported that this essential oil has the potential to reverse the diabetes risk factors through making changes in muscarinic cholinergic system in hippocampus and accordingly can improve memory function (19). According to the mentioned and other conducted studies (14), it seems that nettle is a useful component to treat type 2 diabetes. Fenugreek is another herbal component extensively used to control diabetes, but its efficiency in the control of blood glucose is still unknown. The active components of fenugreek are soluble fiber, saponin of Trigonella foenum-graecum, diosgenin, and 4-hydroxyisoleucine (4-OH-Ile). The blood glucose controlling effects of fenugreek are generally attributed to increasing dietary fiber and saponin (20). 

Animal studies have indicated that the essential oil of fenugreek seed can decrease enzymatic digestion of carbohydrates, gastrointestinal absorption of glucose, and the level of 2hppBG (21). In addition, fenugreek can stimulate glucose absorption in peripheral tissues (22) and secretion of insulin in rats (23). However, according to the aforementioned results, the therapeutic effects of fenugreek are contradictory. Some long-term clinical trials have found that fenugreek can decrease the level of HbA1c, FBS, and 2hppBG (24). According to these studies, it seems that the dose of consumption affects the response to the treatment, and most of the positive therapeutic findings were achieved following the use of higher doses of fenugreek seed powder (> 5 mg) (20).

Cinnamon is another herb with benefits to control blood glucose, though the results have been contradictory. Some studies have indicated its effect on reducing blood glucose in patients with diabetes (25), while some others did not believe in such effects of cinnamon (26). Auto-phosphorylation and -dephosphorylation of insulin receptor, synthesis and transfer of glucose transporter 4 (GLUT-4), regulation of liver metabolism through making changes in pyruvate kinase (PK) and phospho enol pyruvate carboxylase (PEPC), and inhibition of intestinal glucosidase are some of the mechanisms proposed for the effectiveness of cinnamon on blood glucose (27). Hlebowicz et al. indicated the effect of cinnamon on reducing the gastric emptying rate (28). The negative effect of cinnamon on the level of blood urea and uric acid are the points that should be considered in its consumption (29). Nausea, skin rashes, hives, and even seizures following hypoglycemia have been reported following consumption of high doses of cinnamon (27). Garlic (Allium sativum) is an aromatic plant traditionally planted and used in different parts of Iran due to its aroma and healing properties. Although garlic have been under consumption for centuries and in many regions of the world, there is little information about its therapeutic effects. Although animal studies have reported the effect of garlic on reducing blood glucose in diabetic animals, the results in some studies have been contradictory (30). It should be noted that there are a few human studies on the blood glucose reducing effect of garlic (31) and the history of using garlic goes back to thousand years ago. Historically, a great deal of attention has been paid to the role of garlic in reducing cardiovascular risk factors. However, a few scientific data are available to support its therapeutic effects. Recently, the use of garlic to treat diabetes has increased and new information has been reported regarding its blood glucose and lipid-lowering as well as anti-arteriosclerosis effects (32). 

In a study, Rajupadiaet reported that garlic can improve insulin sensitivity, while, metabolic syndrome and oxidative stress reduced the amount of food in rats (33). The study by Jalal et al. entitled: “the blood glucose-lowering effect of shallot and garlic diluted extract”, reported that garlic and shallot extracts had blood-glucose lowering effects on rats in which insulin-resistance was induced by fructose. The shallot extract was more effective in this regard compared to garlic extract (34). In a study by Ebadi et al. entitled: “evaluating the effect of garlic tablet on blood glucose in patients with type 2 diabetes”, the results suggested that use of garlic tablets significantly reduced the level of FBG in the case group, compared to the control. Also, garlic significantly reduced the level of HbA1c (1.5%) in the case group compared to the control group, which was 0.2%. There was no significant reduction in the blood glucose of the control group. In the current study, garlic significantly reduced the level of FBS and HbA1c in the case group compared to the control group. Accordingly, it seems that it can be used as a significant agent to control and treat diabetes.

Walnut, *Junglansregia*, is another plant used in Iranian traditional medicine for its various therapeutic effects such as blood glucose-lowering properties. Walnut leaves include tannin, essential fatty acids, ascorbic acid, flavonoids, folic acid, and para-coumaric acid, in addition to naphthalene derivatives, especially 5-hydroxy-1, and 4-naphthoquinone (35). Juglone is the most abundant compound in different organs of walnut plant with C10H5O2 (OH) formula and 174.16KDa molecular weight. The precursor of juglone, which hydrolysis to juglone, is a glycoside found in combination forms in aerial organs, especially leaves (36). Some previous studies suggested that the brewed walnut leaf and olive can significantly reduce the blood glucose in patients with diabetes (36). 

The effect of hydraulic extract of walnut leaves on healthy and diabetic rats was assessed in another study. It found that dose-dependent consumption of the extract reduced blood glucose in the diabetic rats, but had no effect on their healthy peers. Hence, the effect of such extract is comparable with that of some drugs such as metformin. The study by Hosseini et al. indicated that the level of FBS, HbA1c, total cholesterol, and triglyceride significantly decreased in the patients with diabetes under treatment with walnut, compared with the levels at the beginning of the study and those of the control group (38). 

Black mulberry, Morus nigra. grows on a tree with 4-10 m height, and numerous and short branches. Leaves of black mulberry are dark green with a heart-shaped blade, and irregular and jagged divisions. The skin, root, leaf, and fruit of the tree are used in medicine. The extract of black mulberry leaves has many therapeutic effects such as anti-hypertensive, diuretic, and hypoglycemic effects (39).

Due to the type of drug and its effective amount, the size of the capsules was slightly big and it was difficult for some patients to swallow it.

In conclusion according to the results of the current study, the herbal combination used in the study controlled blood glucose, and considering its HbA1c-lowering effect by 1.31 %, it can be considered an effective medicine to treat diabetes. Since the patients with a history of using OADs as well as reduction of insulin-resistance following the consumption of the herbal medicine in the current study formed the population here, it is recommended to design a study on the early diagnosed cases to evaluate the effectiveness of the herbal combination in the initial treatment of diabetes. 

## List of abbreviations used

CAM: Complementary and alternative medicine; CIM: Complementary and Integrative Medicine; HbA1C:Glycated hemoglobin (hemoglobin A1c); CRP:C reactive protein; FBS: Fasting blood sugar; 2hppBS:2-hour Post-prandial blood sugar ;LDL: Low-density lipoprotein; HDL: High-density lipoprotein; SGOT: Aspartate transaminase; SGPT: Alanine transaminase
